# Intergenerational transmission of body mass index and associations with educational attainment

**DOI:** 10.1186/s12889-022-13270-1

**Published:** 2022-05-04

**Authors:** Hekmat Alrouh, Elsje van Bergen, Eveline de Zeeuw, Conor Dolan, Dorret I. Boomsma

**Affiliations:** 1 Department of Biological Psychology, Faculty of Behavioral and Movement Sciences, Vrije Universiteit Amsterdam, Van der Boechorststraat 7-9, Room MF-H557, 1081 BT Amsterdam, The Netherlands; 2 Amsterdam Public Health Research Institute, Amsterdam, Netherlands; 3 Research Institute LEARN!, VrijeUniversiteit Amsterdam, Amsterdam, Netherlands; 4 Amsterdam Reproduction & Development Research Institute, Amsterdam, Netherlands

**Keywords:** Intergenerational transmission, Educational attainment, Body mass index, Spouse correlation, Structural equation modeling

## Abstract

**Background:**

Individual differences in educational attainment (EA) and physical health, as indexed by body mass index (BMI), are correlated within persons and across generations. The present aim was to assess these associations while controlling for parental transmission.

**Methods:**

We analyzed BMI and EA obtained for 8,866 families from the Netherlands. Data were available for 19,132 persons, including 6,901 parents (mean age 54) and 12,234 of their adult offspring (mean age 32). We employed structural equation modeling to simultaneously model the direct and indirect transmission of BMI and EA from parents to offspring, spousal correlations, and the residual within-person BMI-EA association and tested for gender differences in the transmission parameters.

**Results:**

We found moderate intergeneration transmission for BMI (standardized beta ~ .20) and EA (~ .22), and substantial spousal correlations for BMI (.23) and EA (.51). Cross-trait parent to offspring transmission was weak. The strength of transmission was largely independent of parent or offspring gender. Negative within person EA-BMI correlations were observed for all family members (fathers, -0.102; mothers, -0.147; sons, -0.154; daughters, -0.173). About 60% of the EA-BMI correlation in offspring persisted after taking into account the intergeneration transmission.

**Conclusions:**

The intergenerational transmission for BMI and EA is mainly predictive within traits. Significant spousal and within person correlations in the parental generation are responsible for the effect of parental EA on offspring BMI. Offspring EA and BMI are further correlated beyond parental influences.

**Supplementary Information:**

The online version contains supplementary material available at 10.1186/s12889-022-13270-1.

## Introduction

Body Mass Index (BMI) is an important marker of overall health and is strongly associated with the risk of – and mortality from – chronic diseases [1]. In the last three decades, the prevalence of obesity has dramatically increased globally [2], and the burden of disease attributable to high BMI has more than doubled, although this trend differs across countries [3]. Within countries, overweight and obesity are not randomly distributed across the population, but tend to be more frequent among people with a lower educational attainment (EA) [4] and in low socio-economic strata [5].

One possible explanation for the association between EA and obesity is that a higher EA leads to better occupations, higher income, and higher socioeconomic status (SES). In fact, many studies use EA – as well as occupation and income – either as proxy for, or a component of, SES measures. SES is inversely related to obesity risk in global north countries [6–9]. Higher education and socioeconomic status result in a better health knowledge, and provide the individual with resources that afford healthy food options [10] and free time to engage in physical exercise [11], i.e., behaviors associated with lower obesity risk. In addition, early research showed a residual link between obesity and cognitive deficiency, even after controlling for parental social class [12]. Conversely, obesity itself can influence EA [13], employment [14], future income [15], and other SES measures [16, 17].

EA and BMI are both subject to intergenerational transmission. The average correlation between parent and offspring EA in Western Europe and USA is 0.39 [18]. As for BMI, a meta-analysis of family studies on BMI transmission found a mean parent–offspring correlation of 0.19 [19], while another meta-analysis showed that having a single parent with obesity is strongly associated with childhood obesity (average odds ratio of 3.49)[20].

In addition to the effect of parental BMI on offspring BMI, studies have shown significant associations between parental EA and offspring BMI [21–23]. Of these studies, only one included parental BMI as a covariate, with the finding that only maternal education had a significant effect [21]. Furthermore, many studies have shown significant associations between parental SES and offspring BMI, yet none of these studies controlled for parental BMI [24–30]. When including parental BMI, it is important to account for spousal correlation, or non-random mating. When spouse choice is not random with regard to a particular phenotype, but reflects resemblance between spouses that is higher that would be expected by chance, then the correlation between the phenotype of fathers and mothers needs to be modelled to avoid confounding. Overlooking spousal correlation where one exists can result in overestimating the association between one parent and offspring.

As we have laid out, BMI and EA are intertwined through various processes, and both are subject to intergenerational transmission. Prior studies have generally suggested that parental EA has a distinctive effect on the BMI of offspring, with similar findings concerning parental SES and offspring BMI. We note that these studies did not account for the correlation between parental EA and parental BMI on one hand, and parental BMI and offspring BMI on the other hand. Therefore, we conjecture that these findings—broadly stated as children growing up in low EA households being more susceptible to obesity—may be confounded by parental BMI. Simply put, it is possible that the observed association is due to the fact that EA and BMI are correlated within an individual, and the transmission of both these traits from one generation to another might give the appearance of an association between one trait (EA) in the parent generation with the other (BMI) in the offspring generation. Therefore, we utilized structural equation modeling (SEM) to examine these associations while correcting for the suspected confounding. SEM is a powerful multivariate statistical technique that allows us to test both direct and indirect effects of hypothesized causal relationships among variables [31]. Its utility to our research question lies in its ability to account for the correlation between two causal variables in a regression analysis. The SEM approach also allows us to estimate the residual correlations between EA and BMI in the offspring generation, so that we can assess the residual association while controlling for parental transmission. This examination is important to assess the extent to which EA and BMI associations in the children are independent of the parental effects.

## Methods

### Participants

Participants were registered with the Netherlands Twin Register (NTR). The NTR collects longitudinal data on health, personality and lifestyle from twin families in the Netherlands. These families were recruited by NTR across the Netherlands through city councils, newsletters, and the NTR website. The present study is based on data collected between 2009 and 2012. Participants first received a written invitation including a link to a webpage, where they can log on to a web-based version of the survey with a unique, personal login name and password. If subjects did not access the web-based survey within the 6 weeks after the invitation, they received a paper version of the survey. Between 3–9 months after the paper versions of the survey were sent, subjects who had not responded received a reminder by post, or a reminder by email, if an email address was available. Several groups of non-responders (e.g. twins from incomplete twin pairs) were reminded by phone call [32]. We selected nuclear family members (parents and offspring), resulting in a sample of 19,135 participants. The survey was collected in two versions, a long and a short version. The short version, filled out by 3,421 participants, did not include questions concerning EA. Our sample comprises twins registered with NTR and their parents and siblings. We refer to the twins and their siblings as the offspring generation. There are 12,234 offspring aged between 16 and 97 years and 6,901 parents (2,817 fathers and 4,084 mothers) aged between 21 and 94 years. Note that the age distributions overlap, as twins registered with the NTR include younger twins, often registered with their parents and older twins, usually registered without their parents. A flowchart of the selection process can be found in supplementary figure S1.

The total number of families was 8,866 with an average of 1.4 offspring per family, where 18% of the families included only members of the parental generation. In addition, 45% of families included twins without their parents; 18% included twins and 1 parent; and 19% included twins and 2 parents (see supplementary table S1). There is information for both parent and offspring generation on BMI in 3,214 families (with *N* = 10,650), and on EA in 1,089 families (with *N* = 3,563) (see supplementary tables S2 and S3). EA from participants below the age of 25 years was not included, as they may not have yet reached their highest educational level, resulting in a sample size of 12,078 for EA. The data were checked for outliers and 5 individuals were excluded due to extreme values for height or weight.

### Measures/Phenotyping

BMI was based on self-reported height and weight, and was analyzed as a continuous variable. EA was self-reported and categorized according to the highest achieved educational level. In comparison to the one-dimensional progression that characterizes most secondary and tertiary educational systems around the world, the Dutch educational system is relatively complex, making the number of years of schooling alone insufficient to accurately capture educational level. The system is composed of two fundamental paths, namely a more vocational track (oriented towards manual occupation) and more general track. We classified the reported educational levels and ordered them according to academic performance requirements, associated SES, and projected income and occupational prestige associated with each level [33]. The resulting continuous EA scale has 8 levels (see Table 1). EA and BMI were adjusted for age of participant at the time of completing the survey. Age adjusted measures were used in the analyses. Outlying BMI data were checked against measurements obtained for the same subject from other surveys in the NTR database. Participants were grouped by sex in the analysis, and separate means were estimated for fathers, mothers, sons and daughters.

**Table 1:** Age, BMI, and EA in parents and offspring

		Fathers *n* = 2,817	Mothers *n* = 4,084	Sons *n* = 3,999	Daughters *n* = 8,235
Age	N available data	2,817	4,084	3,999	8,235
	mean	55.88	53.01	31.64	31.91
	SD	7.90	8.04	14.74	14.35
BMI	N available data	2,758	3,960	3,865	7,924
	mean	26.10	25.66	23.53	23.06
	SD	3.27	4.46	3.49	3.84
EA	N available data	2,030	2,897	1,902	4,163
EA level	Dutch equivalent				
Elementary school	Basisschool	1.7%	2.3%	1.1%	1.3%
Lower vocational education	Vmbo/vocational stream	13.2%	11.3%	7.5%	%6.3
Lower general secondary school	Mulo, mavo, vmbo/theoretical stream	9.1%	19.5%	6.1%	9.2%
Intermediate vocational education	Mbo	20.0%	23.1%	20.8%	27.3%
Upper general secondary school	Havo, hbs, atheneum, gymnasium	5.3%	8.7%	2.9%	4.9%
Higher vocational education	Hbo	27.9%	25.5%	29.3%	29.1%
University degree	Post-hbo degree	18.8%	8.9%	25.7%	18.4%
Post-graduate degree	PhD degree	4.1%	0.8%	6.6%	3.6%

*Abbreviations*: *BMI* Body mass index, *EA* Educational attainment, *SD* Standard deviation

### Statistical analyses

**Fig. 1:**
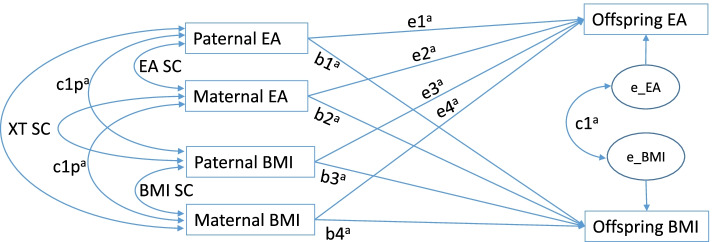
Full model outline. Abbreviations: BMI, body mass index; EA, educational attainment; XT, cross-trait; SC, spousal correlation. ^a^ separate coefficients for each offspring gender

The steps included in SEM first involve model specification, i.e., specifying the hypothesized relationships between the variables, as outlined in Fig. 1. We chose full information (a.k.a. raw data) maximum likelihood (FIML) estimation to obtain estimates of the parameters (and standard errors), and the overall fit (i.e., χ^2^, RMSEA) (Fan et al., 2016) of the model. The parameter estimates lead to an expected covariance matrix that is the most likely one given the observed data. The overall model fit measures provide information on how well our theoretical model fits the data.

We applied SEM to study the phenotypic transmission of BMI and EA from fathers and mothers to their offspring in a large population cohort from the Netherlands. Our model allows us to take into account the correlation of BMI and EA within and between parents, while examining the simultaneous transmission of BMI and EA. The latter includes the prediction of offspring BMI and EA from parental BMI and EA within (e.g., BMI to BMI) and across traits (e.g., BMI to EA). We tested whether the strength of the associations depend on the sex of the parent and the offspring. We examined direct effects, defined as the transmission from parent to offspring within or across traits, and indirect effects, resulting from 1) the correlations between EA and BMI within each parent and 2) the spousal correlations between parents for these traits. For instance, maternal EA has a direct effect path on offspring BMI, an indirect effect path through maternal BMI proportional to the EA-BMI correlation in the mother, and two indirect effect paths through paternal EA and BMI, given non-zero spousal correlations. We included a maximum of 6 offspring (3 males and 3 females) per family. We decided to limit the analysis to a maximum of 6 offspring because of computational considerations. This did not result in an appreciable loss of data: we analyzed 99.32% of the original study sample.

Model specifications: Paternal and maternal transmissions to sons and daughters were specified separately, i.e., we estimated 4 sets (mother-daughter, mother-son, father-daughter, and father-son) of 4 coefficients (EA to EA, BMI to BMI and the cross-transmission coefficients). The following parameters were specified, with estimates for offspring of the same gender (with a maximum of 3 sons and 3 daughters per family) constrained to be equal:

- 16 parent–offspring transmission parameters: 2 traits (EA & BMI) × 2 types of transmission (within- and cross-trait) × 2 parents × 2 offspring genders
- 4 spousal correlations: 2 measures (EA & BMI) × 2 types of correlation (within- and cross-trait)
- 2 within person EA/BMI correlations in the parental generation: fathers and mothers
- 2 residual EA/BMI correlations in the offspring generation: sons and daughters
- 4 variances in the parental generation: 2 traits × 2 genders (fathers and mothers)
- 4 residual variances in the offspring generation: 2 traits × 2 genders (sons and daughters)

We estimated all intercepts for EA and BMI (father, mother, son, daughter) and the within and across-trait correlations for all residuals, i.e. the part in offspring BMI and EA that cannot be ascribed to parental transmission. All correlations were estimated without constraints and thus included 15 within-trait sibling correlations for EA and for BMI and 30 EA/BMI cross-trait correlations, as we allowed for 6 offspring per family.

Thus, in the full model (Fig. 1), all coefficients were estimated separately for sons and daughters and for mothers and fathers. Subsequently we imposed equality constraints on transmission parameters across genders of parents and offspring to test the differences in influence across various parent–offspring gender combinations. For these tests, we applied a Bonferroni correction. Given a family-wise α of 0.01 and 8 tests, the test-wise alpha was 0.01/8 = 0.00125. As mentioned above, we used FIML estimation to fit the model to the data. An advantage of FIML is that it handles missing data optimally: it exploits all available data and is more efficient than list- or pairwise deletion. Frequency tables and descriptive statistics were obtained from IBM SPSS (version 26). We carried out the structural equation modeling in the Lavaan package (version 0.6–6) in R (version 3.6.1).

## Results

### Descriptive statistics

**Fig. 2:**
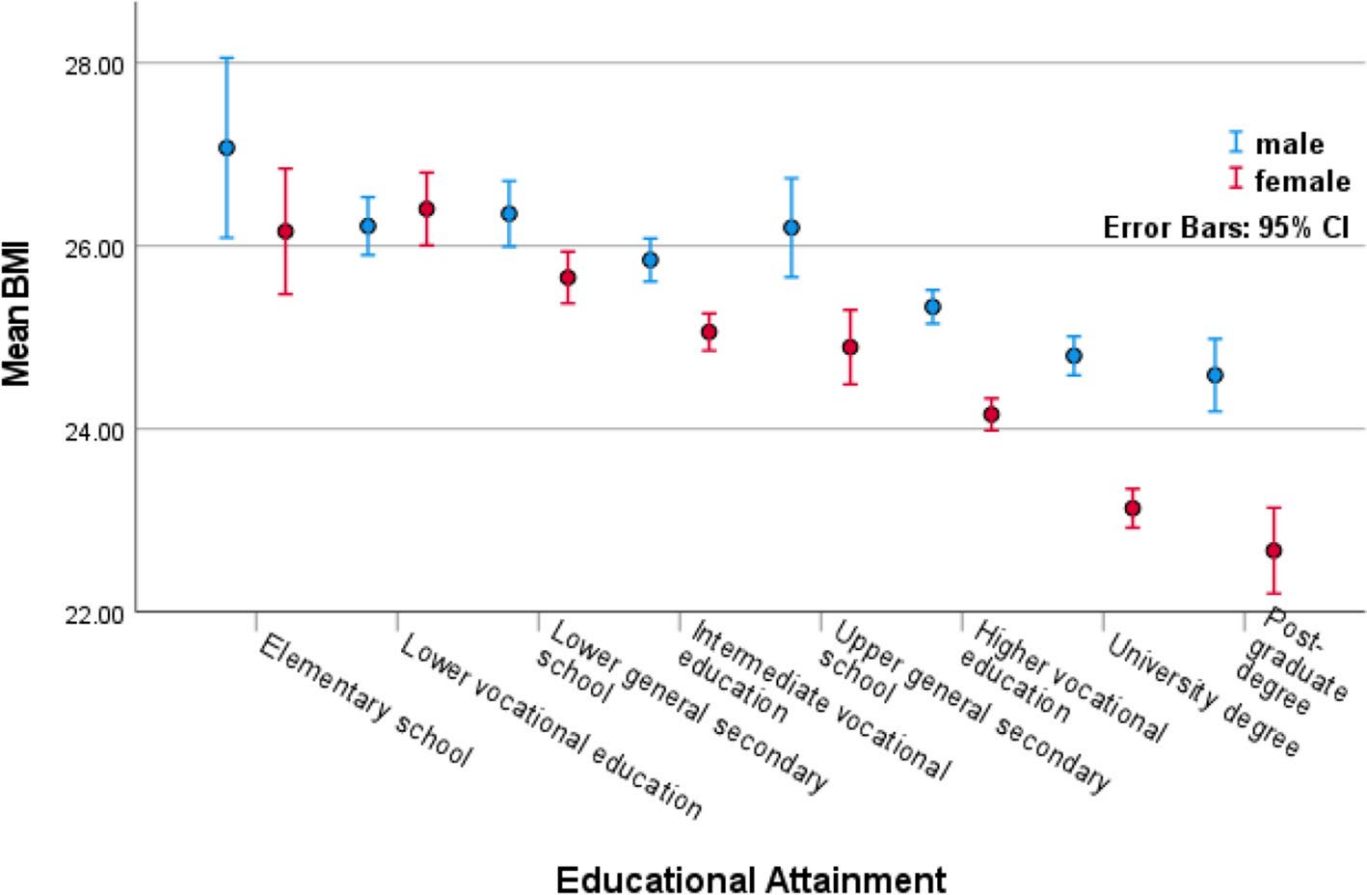
Mean BMI by EA level clustered by sex. Clusters: (male/female). Error bars: 95% confidence intervals

Mean age of fathers was slightly higher than mothers (56 vs. 53 years), while offspring of both genders were both around 32 years. In both generations, the average male BMI was 0.5 points higher than the average female BMI. Mean BMI in parental generation was 26.1 for fathers and 25.6 for mothers, and in offspring generation it was 23.5 for sons and 23.1 for daughters. Correcting for age reduced the differences in BMI between the two generations (i.e., males: 2.6, females: 2.5) to 0.07 BMI points for males and 0.5 for females. Supplementary Figure S2 shows BMI distribution across age for males and females, with higher BMI in older individuals, and consistently higher male BMI in all age groups. EA levels were higher in males than in females, although the difference was smaller in the offspring generation. Figure 2 shows unadjusted mean BMI for each EA level clustered by sex, with higher EA levels associated with lower BMI, especially among females. Supplementary Figure S3 shows EA distribution across age for males and females, with higher levels of education and a narrowing gender gap for younger individuals. The variance of BMI (age adjusted) was larger in females than in males in the parental generation (19.63, CI 18.78–20.48 vs 10.49, CI 9.95–11.03), and in the offspring generation (13.27, CI 12.85–13.68 vs 9.30, CI 8.88–9.71) (Table 2). The variance of EA (age adjusted) was slightly larger in males than in females in the parental generation (3.35, CI 3.15–3.55 vs 2.59, CI 2.46–2.72), and in the offspring generation (2.83, CI 2.65–3.01 vs 2.33, CI 2.23–2.43). Negative within person EA-BMI correlations were observed for all family members (fathers, -0.102; mothers, -0.147; sons, -0.154; daughters, -0.173). Unadjusted correlations (see supplementary table S4) tended to be higher for most measures compared to age adjusted correlations.

**Table 2:** Observed covariance and correlation table for age adjusted EA and BMI

	Offspring BMI (male/female)	Offspring EA (male/female)	Paternal BMI	Maternal BMI	Paternal EA	Maternal EA
Offspring BMI (male/female)	9.298/13.268	-0.791/-0.965	1.804/2.264	1.927/3.432	-0.405/-0.434	-0.269/-0.416
Offspring EA (male/female)	-0.154/-0.173	2.796/2.329	-0.384/-0.436	-0.837/-0.410	0.860/0.629	0.597/0.651
Paternal BMI	0.183/0.192	-0.071/-0.088	10.492	3.277	-0.606	-0.583
Maternal BMI	0.143/0.213	-0.112/-0.061	0.228	19.630	-1.402	-1.050
Paternal EA	-0.073/-0.065	0.279 /0.225	-0.102	-0.173	3.350	1.509
Maternal EA	-0.055/-0.071	0.220/0.265	-0.112	-0.147	0.512	2.593

Upper triangle, covariance. Lower triangle: correlation. Diagonal: variance

*Abbreviations*: *BMI* Body mass index, *EA* Educational attainment

### Direct and indirect effects, full model

**Fig. 3:**
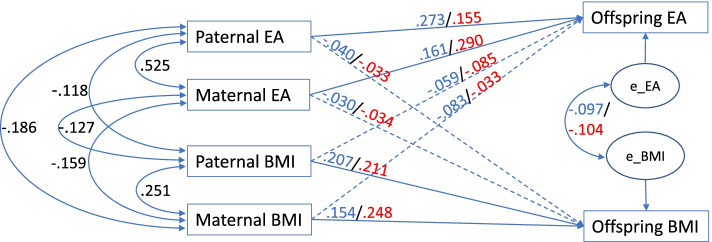
Full model results. Standardized transmission coefficients and correlations (male/female offspring). Solid lines: statistically significant at *p* < 0.01. Dashed lines: statistically insignificant. Abbreviations: BMI, body mass index; EA, educational attainment

Table 3 summarizes results for the full model, which are summarized in Fig. 3. The overall goodness of fit of the full model was acceptable (see supplementary table S7). A direct effect was defined as the transmission coefficient in the regression of parental BMI/EA on offspring BMI/EA. An indirect effect was calculated as the sum of three paths through correlations of one parental measures with the remaining three. For example, if we consider paternal BMI, there is one direct path and three indirect paths going through maternal BMI, paternal EA, and maternal EA. Total effects were calculated by adding up direct and indirect effects. In the full model, within-trait direct effects (parental BMI on offspring BMI and parental EA on offspring EA) were positive and significant for both parents, while cross-trait direct effects were insignificant, i.e., parental BMI did not have significant direct effects on offspring EA, and vice versa. Indirect effects were larger than direct effects for cross-trait transmission, due to significant cross-trait correlations at the parental level (see Table 2). For example, the standardized direct effect of maternal EA on male offspring BMI was -0.030, while the indirect effect was -0.072, adding to a total effect of -0.101). Residual within person EA—BMI correlations in the offspring generation remained significant after accounting for parental effects, which indicates EA and BMI are still associated after controlling for parental influence. To check if extreme BMI values (due to disorders such as anorexia nervosa or monogenic causes of morbid obesity) influenced the results, the analysis was repeated excluding 25 subjects with BMI values less than 15 or higher than 45. Parameter estimates obtained in the reduced sample hardly differed from those obtained in the full sample.

**Table 3:** Associations between Parent/Offspring BMI/EA (full model, see Fig. 1)

	Raw			Standardized				
	Direct			Direct			Indirect	Total
**Sons**								
**BMI**	Estimate	Lower CI	Upper CI	Estimate	Lower CI	Upper CI	Estimate	Direct + indirect
Paternal BMI	0.192*	0.140	0.244	0.207	0.151	0.263	0.047	0.254
Maternal BMI	0.105*	0.069	0.141	0.154	0.101	0.207	0.064	0.218
Paternal EA	-0.066	-0.195	0.064	-0.040	-0.119	0.039	-0.068	-0.108
Maternal EA	-0.055	-0.191	0.081	-0.030	-0.102	0.043	-0.072	-0.101
**EA**								
Paternal EA	0.247*	0.151	0.343	0.273	0.167	0.380	0.107	0.381
Maternal EA	0.165*	0.058	0.272	0.161	0.057	0.265	0.164	0.326
Paternal BMI	-0.030	-0.084	0.025	-0.059	-0.166	0.049	-0.074	-0.132
Maternal BMI	-0.031	-0.064	0.002	-0.083	-0.173	0.006	-0.091	-0.175
**EA-BMI residual correlation**	-0.405*	-0.663	-0.148	-0.097	-0.149	-0.044		
**Daughters**								
**BMI**	Estimate	Lower CI	Upper CI	Estimate	Lower CI	Upper CI	Estimate	Direct + indirect
Paternal BMI	0.235*	0.183	0.287	0.211	0.164	0.258	0.071	0.282
Maternal BMI	0.202*	0.168	0.236	0.248	0.206	0.289	0.065	0.313
Paternal EA	-0.065	-0.188	0.059	-0.033	-0.095	0.030	-0.089	-0.122
Maternal EA	-0.077	-0.203	0.048	-0.034	-0.090	0.022	-0.083	-0.118
**EA**								
Paternal EA	0.127*	0.054	0.200	0.155	0.066	0.243	0.168	0.323
Maternal EA	0.271*	0.199	0.344	0.290	0.212	0.367	0.097	0.387
Paternal BMI	-0.040	-0.077	-0.003	-0.085	-0.165	-0.006	-0.063	-0.148
Maternal BMI	-0.011	-0.035	0.013	-0.033	-0.104	0.039	-0.096	-0.129
**EA-BMI residual correlation**	-0.490*	-0.692	-0.289	-0.104	-0.144	-0.065		

Abbreviations: *BMI* body mass index; *EA* educational attainment; *CI* confidence interval

*p<0.01

### Gender differences in transmission parameters

To assess gender differences in transmission from fathers and mothers to sons and daughters, we imposed equality constraints on transmission coefficients, and tested these constraints by likelihood ratio tests. We found significant differences only for maternal BMI on offspring BMI (supplementary table S5), where the direct effect was almost double in females (males: b = 0.105 CI 0.061, 0.148; females: b = 0.202 CI 0.161, 0.243). Similar analyses for parental gender showed no significant differences between paternal and maternal transmission coefficients (supplementary table S6), i.e., transmission from fathers and mothers were of equal magnitude. Based on the results of these tests, we arrived at model 2. Parameter estimates for this model, shown in Table 4, indicate small, but significant, direct effects for cross-trait transmission from parents to offspring. Standardized within-trait transmission coefficients were similar for both EA and BMI, and generally did not depend on the gender of the parent or offspring, with the exception of maternal BMI on offspring BMI. Cross-trait transmission coefficients were larger for offspring BMI than offspring EA, i.e., parental EA had a larger effect on offspring BMI than parental BMI had on offspring EA. Finally, the within trait spousal correlations were 0.228 for BMI, and 0.512 for EA. Cross-trait spousal correlations were -0.112 for maternal EA/paternal BMI, and -0.173 for paternal EA/maternal BMI. Both models had good model fit measures, with model 2 scoring slightly higher than the full model (e.g. x^2^: 59.5 vs 52.1; degrees of freedom: 51 vs 44) with a likelihood ratio test *p*-value = 0.39, indicating no significant difference between the two models. Model fit measures are presented in supplementary table S7.

**Table 4:** Associations between Parent/Offspring BMI/EA (model 2)

	Raw			Standardized			
				Sons		Daughters	
**Offspring BMI**	Estimate	Lower CI	Upper CI	Paternal	Maternal	Paternal	Maternal
Paternal BMI (sons)	0.212*	0.174	0.251	0.228	-	-	-
Paternal BMI (daughters)	0.212*	0.174	0.251	-	-	0.191	-
Maternal BMI (sons)	0.098*	0.064	0.133	-	0.145	-	-
Maternal BMI (daughters)	0.209*	0.177	0.241	-	-	-	0.257
Parent EA	-0.066*	-0.107	-0.026	-0.040	-0.035	-0.034	-0.029
**Offspring EA**							
Parent EA	0.201*	0.177	0.226	0.224	0.197	0.244	0.215
Parent BMI	-0.024*	-0.039	-0.009	-0.047	-0.064	-0.051	-0.070

*Abbreviations*: *BMI* Body mass index, *EA* Educational attainment, *CI* Confidence interval

^*^p < 0.005

## Discussion

Offspring BMI was negatively correlated with parental EA (*r* ~ -0.07) but this correlation was low and insignificant in the full model, when accounting for parental BMI. This suggests that the effect of parental EA on offspring BMI is mainly mediated through parental BMI. Similar trends were seen when examining the influence of parental factors on offspring EA. The significant lowering of parental EA/BMI regression coefficients, when controlling for the other parental traits, supports earlier findings of shared factors influencing EA and BMI. This finding calls into question the general consensus that parental EA – and subsequently parental SES – has a direct effect on the odds of developing obesity in the offspring generation. Rather, it is an individual’s own EA that has a higher association with their obesity risk. Potential mechanism that explain these associations span a variety of social, behavioral, metabolic, and neurocognitive processes. Individuals with higher BMI are more likely to have lower self-esteem and to experience social marginalization [34, 35]. Behavioral factors such as self-control and delayed gratification are associated with EA [36–38] as well as obesity [39, 40]. Decreased cognitive function is associated with impaired metabolic pathways associated with obesity such as insulin signaling [41] and leptin regulation [42]. Finally, obesity appears to have significant genetic overlap with brain and cognitive measures [43] as well as EA [44].

To our knowledge, this is the first study in an adult population to examine the effects of parental EA on adult offspring BMI, while controlling for parental BMI. The average age of offspring in our sample is 32 years, which means that most will have left the parental home around 10 years earlier, as the average age at which offspring leave home in the Netherlands is 22.7 years for daughters and 24.2 for sons [45]. Still, we observe parental BMI to be correlated with their adult offspring BMI (*r* = 0.18). This is in line with a literature review of studies examining parent–offspring BMI associations [19]. Age adjusted parent–offspring BMI correlations were lower after accounting for parental EA, which shows that a portion of intergenerational BMI transmission is due to factors related to parental EA.

Within-person EA-BMI correlations were small in the offspring generation before accounting for parental factors (*r* = -0.15 for sons and -0.17 for daughters), with similar level for mothers (-0.15) and slightly lower for fathers (-0.10). In the offspring generation, these correlations were -0.10 after controlling for parental transmission. This persistence of association suggests that the relationship between EA and BMI is largely independent of parental factors, which implies that interventions aimed at improving EA (and consequently SES) can translate into desirable changes in BMI, irrespective of parental EA and BMI.

Gender did not play a significant moderating role in our model. Transmission coefficients were largely similar from fathers and mothers to sons and daughters, with the exception of BMI transmission from mothers to sons, which was lower than other parent–offspring combinations, but had the same direction of association (i.e. positive). The absence of gender differences confirms findings of prior studies [20], although few studies have reported differences between fathers and mothers in BMI transmission [46, 47].

Parents in our sample exhibited moderate levels of spousal correlation for BMI. For EA, the association was high. The observed spousal correlation for BMI in our study (*r* = 0.23) is somewhat higher than that reported in most other studies, averaging at 0.15 [48]. Increased rates of spousal correlation over birth cohorts have been hypothesized to have contributed to the rise in obesity prevalence [49]. Indeed, the odds of offspring obesity increase markedly when both parents have obesity [50]. From our study design, it is unclear whether this correlation existed prior to marriage/cohabitation (due to phenotypic assortment or social homogamy) or developed with time (due to marital interaction). However, the latter scenario would involve an increase over time in spousal correlation for BMI, which is generally not found [48].

The main strengths of this study are a large sample size and age range for parents and offspring, as well as use of multiple offspring within families. Our study sample covers different geographic areas and socioeconomic classes in the Netherlands. Recruitment of twins – considered representative of the general population [51] – and their families into the NTR was done through multiple channels including city council registries, leading to a sample that is reasonably representative of the Dutch population. Although the study relied on self-reported measures for height, weight, and EA for parents and offspring, the reliability in self-reporting of height and weight is good: in an analysis of a subsample of 6,026 individuals, we observed a correlation of 0.93 between self-reported BMI and BMI measured by a research nurse or assistant [52]. We analyzed BMI as a continuous variable rather than a (clinical) binary variable (obese/overweight vs. normal weight), because we wanted to address the process of transmission as it pertains to the full range of BMI in the general population in the Netherlands. In this approach we assume that the process of transmission as we identify it is relevant to the full range of BMI and EA. We note that it is possible to fit our present model (see Fig. 1) to a binary BMI variable, but this would merely result in a loss of information. Specifically, the analysis in the case of a binary BMI variable is based on the liability threshold model, in which the model is fitted to latent continuous BMI variables. There is no advantage to this compared to fitting the directly to observed continuous BMI variables, but there is the disadvantage of a loss of power associated with loss of information. For this reason, we opted to use BMI as a continuous variable in our analysis.

There are also some limitations to the current study. While BMI is a common, convenient measure of obesity, other methods, such as skin fold thickness and percent body fat from dual energy X-ray absorptiometry, may provide more accurate estimates. In our analyses, we have modeled the process of transmission of EA and BMI from adult parents to adult offspring. Our resulting model is supposed to represents this process as it takes place in the general Dutch population, and with respect to the full range of BMI. As such, this model is based on the assumption that the process is the same regardless of the actual level of BMI in the parents or their offspring. We recognize that it is possible that the transmission process may differ (e.g., in terms of the parameters) in extremes of the BMI distribution, i.e., in the obese and underweight subpopulations. Detecting such heterogeneity is a statistically challenging task, which is beyond our present aim.

Our results pertain to adult offspring who generally have left the parental household. A next step in future research is to examine whether the associations and transmission results based on adult offspring are also seen in younger offspring who still live with their parents, and likely share more of the home environment. This will facilitate the disentangling of the different aspects by which parents influence their offspring’s probability of having overweight or obesity. This is important to inform policy and interventions aimed at reducing the prevalence of these conditions. Our results suggest that improving the SES of a household may not on its own be sufficient to reduce the odds of obesity among offspring. It is imperative that policies focus on long term strategies such as educational expansion and improving social mobility, which would have a more pronounced impact on obesity rates one generation after another.

## Conclusions

Our study assessed the EA-BMI association while controlling for parental transmission and highlights in its results the existence of this association after controlling for parental EA and BMI. We documented significant within trait transmission for both traits, while cross trait transmission became insignificant after controlling for the same trait in the parental generation. This pattern points towards shared factors that influence both measures. Thus, the correlation between EA and BMI within individuals is partially due to parental factors, but the majority portion of correlation is independent of parental influences.

## Supplementary Information

**Additional file 1.**

## Data Availability

The datasets used and/or analyzed during the current study available from the corresponding author on reasonable request. Code for SEM portion of the analysis is available at the following Github repository: • Project name: SEM model of BMI/EA intergenerational transmission with multiple (6) offspring per family • Project home page: https://github.com/hekmatov/EA-BMI-IGT-adult • Archived version: 7978f29 • Operating system(s): Platform independent • Programming language: R • Other requirements: R 3.6.1 or higher. Lavaan package 0.6–6 or higher • License: MIT • Any restrictions to use by non-academics: licence needed

## Abbreviations

EA: Educational attainment
BMI: Body mass index
SES: Socioeconomic status
NTR: Netherlands Twin Register
SEM: Structural equation modeling
FIML: Full information maximum likelihood
