# Supplementary material for: Intergenerational transmission of body mass index and associations with educational attainment
Source: BMC Public Health. 2022 May 4;22:890. doi: 10.1186/s12889-022-13270-1 (PMC9069759; doi:10.1186/s12889-022-13270-1)
Supplement: Supplementary file 1 — Additional file 1. [file 12889_2022_13270_MOESM1_ESM.docx]

**Intergenerational Transmission of Body Mass Index and Associations with Educational Attainment**

Hekmat Alrouh^1,2^ (0000-0003-2224-149X),
Elsje van Bergen^1,2,3^ (0000-0002-5860-5745),
Eveline de Zeeuw^1,2^ (0000-0001-9042-7419),
Conor Dolan^1,2^ (0000-0002-2496-8492),
Dorret I. Boomsma^1,2,4^ (0000-0002-7099-7972)

^1^ Department of Biological Psychology, Faculty of Behavioral and Movement Sciences, Vrije Universiteit Amsterdam, Amsterdam, Netherlands
^2^ Amsterdam Public Health Research Institute, Amsterdam, Netherlands ^3^ Research Institute LEARN!, Vrije Universiteit Amsterdam, Amsterdam, Netherlands
^4^ Amsterdam Research & Development Research Institute, Amsterdam, Netherlands

Contact info of corresponding author:
Hekmat Alrouh, Vrije Universiteit Amsterdam, Dept Biological Psychology
Van der Boechorststraat 7-9, Room MF-H557,
1081 BT Amsterdam, The Netherlands
Tel: + 31 20 598 5923 / Email: [h.alrouh@vu.nl](mailto:h.alrouh@vu.nl)

Supporting Information:

Table S1: Distribution of parents and offspring among families

|  |  | Number of offspring per family | | | | | | | Total |  |
| --- | --- | --- | --- | --- | --- | --- | --- | --- | --- | --- |
|  |  | 0 | 1 | 2 | 3 | 4 | 5 | 6 |  |  |
| Number of parents per family | 0 | 0 | 2239 | 1456 | 232 | 47 | 5 | 7 | 3986 |  |
|  | 1 | 1234 | 726 | 679 | 195 | 20 | 3 | 2 | 2859 |  |
|  | 2 | 346 | 454 | 813 | 363 | 38 | 7 | 0 | 2021 |  |
| Total |  | 1580 | 3419 | 2948 | 790 | 105 | 15 | 9 | 8866 |  |

Table S2: Distribution of body mass index (BMI) for parents and offspring among families

|  |  | Number of offspring with BMI data | | | | | | | Total |
| --- | --- | --- | --- | --- | --- | --- | --- | --- | --- |
|  |  | 0 | 1 | 2 | 3 | 4 | 5 | 6 |  |
| Number of parents with BMI data | 0 | 133 | 2272 | 1400 | 216 | 42 | 6 | 5 | 4074 |
|  | 1 | 1238 | 746 | 668 | 182 | 19 | 2 | 2 | 2857 |
|  | 2 | 340 | 476 | 764 | 315 | 34 | 6 | 0 | 1935 |
| Total | | 1711 | 3494 | 2832 | 713 | 95 | 14 | 7 | 8866 |

BMI: body mass index

Table S3: Distribution of educational attainment (EA) for parents and offspring among families

|  |  | number of offspring with EA data | | | | | | | Total |
| --- | --- | --- | --- | --- | --- | --- | --- | --- | --- |
|  |  | 0 | 1 | 2 | 3 | 4 | 5 | 6 |  |
| Number of parents with EA data | 0 | 2731 | 1386 | 1008 | 177 | 39 | 6 | 6 | 5353 |
|  | 1 | 1573 | 255 | 197 | 64 | 6 | 3 | 1 | 2099 |
|  | 2 | 851 | 239 | 205 | 102 | 15 | 2 | 0 | 1414 |
| Total | | 5155 | 1880 | 1410 | 343 | 60 | 11 | 7 | 8866 |

EA: educational attainment

Table S4: Raw covariance and correlation table (unadjusted for age).

|  | Offspring BMI (male/female) | Offspring EA (male/female) | Paternal BMI | Maternal BMI | Paternal EA | Maternal EA |
| --- | --- | --- | --- | --- | --- | --- |
| Offspring BMI (male/female) | 12.177/  14.778 | -1.342/  -1.472 | 2.019/  2.347 | 2.243/  3.580 | -0.728/  -0.621 | -0.789/  -0.693 |
| Offspring EA (male/female) | -0.220/  -0.230 | 3.053/  2.759 | -0.586/  -0.739 | -0.954/  -0.773 | 0.897/  0.623 | 0.628/  0.603 |
| Paternal BMI | 0.178/0.188 | -0.103/-0.137 | 10.536 | 3.303 | -0.629 | -0.591 |
| Maternal BMI | 0.145/0.210 | -0.123/-0.105 | 0.230 | 19.593 | -1.419 | -1.110 |
| Paternal EA | -0.114/-0.0880 | 0.279/0.204 | -0.105 | -0.175 | 3.374 | 1.529 |
| Maternal EA | -0.138/-0.110 | 0.219/0.222 | -0.111 | -0.153 | 0.508 | 2.684 |

Upper triangle, covariance. Lower triangle: correlation. Diagonal: variance.
Abbreviations: BMI, body mass index; EA, educational attainment

Table S5: Chi-square (likelihood ratio) test results for path model fit of transmission coefficients equality constraint across male and female offspring (see figure 1).

|  | Constrained coefficients | df | x^2^ | x^2^ diff | df diff | p (>x^2^) |
| --- | --- | --- | --- | --- | --- | --- |
| Full model | - | 44 | 52.132 | - | - | - |
| All paths (Omnibus test) | e3, e4, e5, e6, b3, b4, b5, b6 | 52 | 84.836 | 32.704 | 8 | 0.0001 |
| P EA → O EA | e3 | 45 | 56.303 | 4.171 | 1 | 0.0411 |
| M EA → O EA | e4 | 45 | 54.999 | 2.868 | 1 | 0.0904 |
| P BMI → O EA | e5 | 45 | 52.230 | 0.099 | 1 | 0.7534 |
| M BMI → O EA | e6 | 45 | 53.135 | 1.003 | 1 | 0.3165 |
| P EA → O BMI | b3 | 45 | 52.132 | 0.000 | 1 | 0.9929 |
| M EA → O BMI | b4 | 45 | 52.192 | 0.060 | 1 | 0.8062 |
| P BMI → O BMI | b5 | 45 | 53.551 | 1.420 | 1 | 0.2335 |
| M BMI → O BMI | b6 | 45 | 68.168 | 16.037 | 1 | 0.0001* |

Abbreviations: df, degrees of freedom; x^2^, chi-square; diff, difference; P, paternal; M, maternal; EA, educational attainment; BMI, body mass index. *significant test after Bonferroni correction (p<0.00125)

| Constrained path | Constrained coefficients | Df | x^2^ | x^2^ diff | df diff | p (>x^2^) |  |
| --- | --- | --- | --- | --- | --- | --- | --- |
| Full model | - | 44 | 52.132 | - | - | - |  |
| All paths (Omnibus test) | e3=e4, e5=e6, b3=b4, b5=b6 | 52 | 65.019 | 12.887 | 8 | 0.1632 |  |
| P EA → O EA (m) | e3m = e4m | 45 | 52.863 | 0.732 | 1 | 0.3591 |  |
| P EA → O EA (f) | e3f = e4f | 45 | 56.570 | 4.438 | 1 | 0.0253 |  |
| P BMI → O EA (m) | e5m = e6m | 45 | 52.133 | 0.001 | 1 | 0.9707 |  |
| P BMI → O EA (f) | e5f = e6f | 45 | 53.417 | 1.286 | 1 | 0.3148 |  |
| P EA → O BMI (m) | b3m = b4m | 45 | 52.139 | 0.007 | 1 | 0.9355 |  |
| P EA → O BMI (f) | b3f = b4f | 45 | 52.144 | 0.012 | 1 | 0.9166 |  |
| P BMI → O BMI (m) | b5m = b6m | 45 | 57.699 | 5.567 | 1 | 0.0587 |  |
| P BMI → O BMI (f) | b5f = b6f | 45 | 52.963 | 0.832 | 1 | 0.4044 |  |

Table S6: Chi-square (likelihood ratio) test results for path model fit of transmission coefficients equality constraint across fathers and mothers (see figure 1).

Abbreviations: df, degrees of freedom; x^2^, chi-square; diff, difference; P, parent; O, offspring; EA, educational attainment; BMI, body mass index; m, male offspring; f, female offspring.

Table S7: model fit measures

|  | model 1 (full model) | model 2 (parsimonious) |
| --- | --- | --- |
| χ^2^ | 52.132 | 59.469 |
| df | 44 | 51 |
| cfi | 0.99762 | 0.99776 |
| rmsea | 0.00458 | 0.00402 |
| rmsea lower ci | 0 | 0 |
| rmsea upper ci | 0.00886 | 0.00811 |
| srmr | 0.02377 | 0.02721 |

Abbreviations: χ^2^, Chi-Square Statistic; df, degrees of freedom; ci, confidence interval.
cfi: The comparative fit index (CFI) analyzes the model fit by examining the discrepancy between the data and the hypothesized model, while adjusting for the issues of sample size inherent in the chi-squared test of model fit, and the normed fit index. CFI values range from 0 to 1, with larger values indicating better fit.
rmsea: root mean square error of approximation (RMSEA) is an absolute fit index, which is based on the fact that the fitted model is an approximation to the true model. RMSEA values of 0.01, 0.05 and 0.08 indicate excellent, good and mediocre approximation, respectively.
srmr: Standardized Root Mean Square Residual (SRMR) is defined as the difference between the observed correlation and the model implied correlation matrix. Thus, it allows assessing the average magnitude of the discrepancies between observed and expected correlations as an absolute measure of (model) fit criterion.

Figure S1: Flowchart of subject selection process

Twin family members:
2,141 siblings + 6,902 parents

Adult twins:
10,178

8,866 families:
12,319 offspring +

6,902 parents

8,866 families:
12,317 offspring +

6,901 parent

Excluded: known or suspected transgender = 3 individuals

Excluded: offspring exceeding 6 per family = 83 offspring

8,866 families:
12,234 offspring +

6,901 parent

Figure S2: Mean BMI by age, all participants
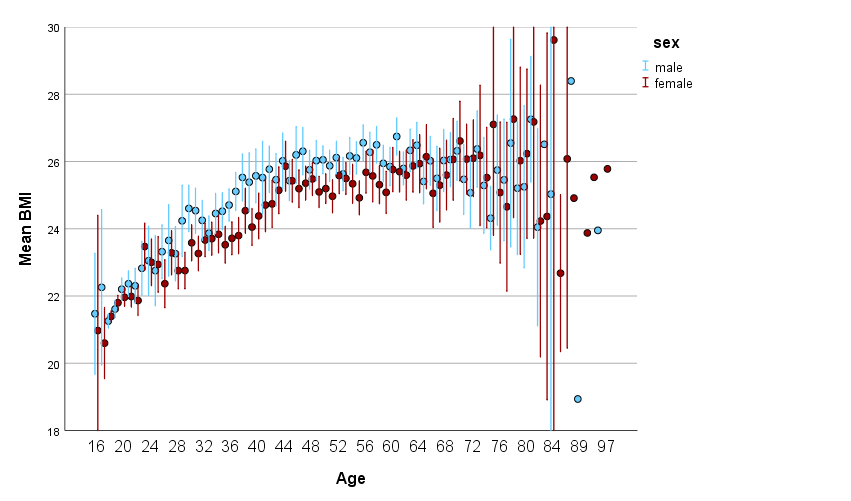

Error bars: 95% confidence intervals. Abbreviations: BMI, body mass index

Figure S3: Mean educational attainment by age, all participants
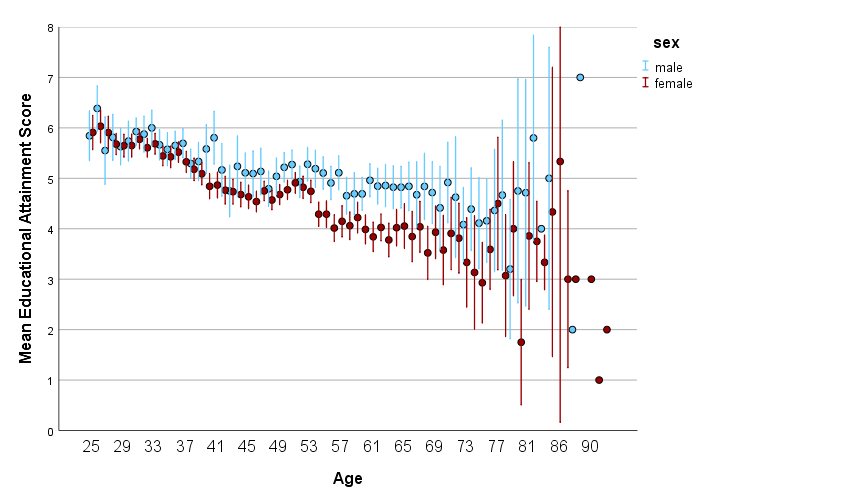

Error bars: 95% confidence intervals
